# What would happen to education if we take education evidence seriously?

**DOI:** 10.1007/s40037-014-0129-9

**Published:** 2014-06-13

**Authors:** C. P. M. van der Vleuten, E. W. Driessen

**Affiliations:** Department of Educational Development and Research, Maastricht University, PO Box 616, 6200 MD Maastricht, the Netherlands

**Keywords:** Education evidence, Education research, Education practice

## Abstract

Educational practice and educational research are not aligned with each other. Current educational practice heavily relies on information transmission or content delivery to learners. Yet evidence shows that delivery is only a minor part of learning. To illustrate the directions we might take to find better educational strategies, six areas of educational evidence are briefly reviewed. The flipped classroom idea is proposed to shift our expenditure and focus in education. All information delivery could be web distributed, thus creating more time for other more expensive educational strategies to support the learner. In research our focus should shift from comparing one curriculum to the other, to research that explains why things work in education and under which conditions. This may generate ideas for creative designers to develop new educational strategies. These best practices should be shared and further researched. At the same time attention should be paid to implementation and the realization that teachers learn in a way very similar to the people they teach. If we take the evidence seriously, our educational practice will look quite different to the way it does now.

## Introduction

Education in the twenty-first century is not that different from education in the eighteenth century. Most education relies heavily on teachers transmitting information to learners. In sharp contrast, the practice of medicine in the twenty first century is quite different from medicine in the eighteenth century. Medicine has evolved substantially through research and the application of research findings into practice. Naturally education has modernized as well. Student-centred education programmes, such as for example problem-based learning, have also evolved considerably. Although these newer approaches receive quite some attention in the scientific media and our education conferences, we would argue that most schools of medicine or other health sciences programmes in university still rely heavily on the lecture as the most dominant form of instruction. In our common conception of learning we assume that information delivery, particularly when stemming from an expert, leads to good learning. With all the evidence on education as we have it, this view on education is rather naive. Our central argument is that learning is not so much a matter of information delivery, but of information processing. Good education provides strategies to facilitate this processing of information. Naturally, information delivery is part of learning, yet only a very limited part. A lot of the processing we simply leave to the learner. We do not really support that part of the learning process.

In our view, education would look very different if we were to take the education evidence seriously. Is that because we have vast evidence that one approach, say problem-based learning, works tremendously better than other approaches? There is some evidence for this, but it is not the kind of evidence we should be looking for. These are questions that are too big to be able to properly answer scientifically. One might also ask about the effectiveness of two health care systems; health care systems are also too complex to be compared meaningfully. This is not to say that it is not possible to assemble data on such large comparisons. Naturally such data have been assembled around problem-based learning (reasonably showing its effectiveness) [1], but there are major methodological problems in these comparisons and it does not answer why problem-based learning works [3]. We need evidence on the building blocks of the larger educational system, preferably also explaining why things work under which conditions [4]. Evidence in education is therefore more than insights from empirical evidence stemming from educational research and other related domains. It also includes theories that are based on empirical evidence. Theories provide explanations why things may work in education.

This paper will take a collection of areas where we have solid evidence on how learning is facilitated. Each of these areas could be a complete review paper, deserving publication in its own right. Here we only sketch the area, just to show in which direction education might be moving. We will finally discuss a strategy with which these directions could be achieved. We deliberately do not discuss evidence on assessment, a topic on which we more regularly publish. We refer to other publications summarizing that area of the literature [5].

## Areas of evidence

### Elaboration

Cognitive science has been a revolution to psychology [6] with massive implications for education and learning. It studies how our memory works and how we store and retrieve information in long-term memory. We will only focus on one element specifically relevant to serve as a contrast to information transmission. Cognitive psychology tells us that learning is facilitated when the learner processes the information. Learning is not so much a matter of consuming information but of active processing of the information by the learner. The term elaboration is used to describe this phenomenon [7, 8]. Elaboration can be done in many various ways. Any activity that allows learners to use the information is useful. There are infinite strategies to promote elaboration. For example, elaboration is facilitated by discussing with others, by schematizing, by summarizing, by verbalizing it in one’s own words, or by applying the information to another situation or problem. Elaboration is hindered when the learner passively consumes the information. In purely traditional lecture-based systems, most elaboration on learning content will not take place in the lecture theatre but elsewhere. In all probability, most elaboration will occur when the learner needs to prepare for the exam at the end of the course. The learner will elaborate by him or herself. We do not offer special support for this, for example certain education strategies that would help the learner to elaborate on learning content. In conclusion, working with information is very important for understanding and learning the information. We should devise educational strategies to promote elaboration as much as possible.

### Cooperative learning

There is a wealth of evidence showing that learning in cooperation with others is much more effective than learning alone [9]. We also know the conditions under which learning with others is most productive. These conditions include equality in participation, members carrying individual responsibility, tasks that require mutual positive interdependence, and finally simultaneous interactions promoting cooperative learning [9]. Learning in groups has some disadvantages as well and comes at a ‘cost’. When certain conditions are met, the benefits outweigh the costs and cooperative learning is very effective. In most traditional educational systems, we highly rely on the individual learner. By not using cooperative learning in some form, we are not making education as effective as it could actually be. Moreover, in later professional life virtually all work is team-based. Working in cooperation with others will therefore not only lead to more effective education, it may also represent a desirable learning outcome: the ability to work with others.

### Feedback

It almost seems like a platitude, but feedback works [10]. There is hardly anything that is more powerful to learning than feedback. We also understand the conditions under which feedback is most effective [10–12]. These include, among others, the credibility of the source, the focus of attention (task oriented rather than person oriented), and the feedback culture in the environment. Another very effective strategy is to create a dialogue around feedback, a dialogue in which follow-up on the feedback is promoted and where help and support is being provided [13].

The effectiveness of feedback for learning may seem a platitude, but we underuse it dramatically in educational practice. A lot of educational practices treat feedback in a rather reductionist way. In most traditional systems the feedback that students receive is often restricted to the outcomes of assessments, often in the form of grades. Grades are in a way the poorest form of feedback one can get [10]. Although there is convincing evidence for the importance of narrative feedback for learning, the same research tells us that it is underused in medical education [14]. Furthermore, in workplace learning feedback is often left implicit or not given at all [15]. The learning of complex skills, a learning outcome pursued by all curricula, can best be achieved by continuous or longitudinal attention with regular feedback and follow-up [16]. Most of our education practices are far removed from that situation.

### Mentoring

We already hinted in the previous paragraph at the value of sharing learning information with someone else. The research around mentoring is really very positive. It is associated with increased use of feedback, improved professional development, career preparation and success (including higher (clinical) production, higher positions) and prevention of production loss such as for example through burnout [17]. In many other domains, such as sports or music [18], enduring personal relationships exist with learners, which are often lacking in our educational training programmes. A longitudinal mentoring relationship will help the feeling of relatedness and competence of learners, and at the same time support the autonomy of students. Relatedness, competence and autonomy are, according to the self-determination theory, conditional for the intrinsic motivation of learners [19]. Mentors should therefore support learners autonomy, e.g. listening to and acknowledging student’s perspectives, enquiring what students want [20]. The task of the mentor is thus primarily coaching, and to ask questions rather than give answers. We could schedule mentoring programmes best when emotions come into play in learning. Transitions are typically moments of emotions and stress [21, 22], as well as inherent emotion-laden learning environments such as workplaces [23]. Or we could use it for those who need it most.

So even though the benefits of mentoring are widely acknowledged, mentoring is largely underused in medical education: less than 50 % of medical students and residents have a mentor. What is even more striking is that underrepresented minority residents and females are less likely to have a mentor than their peers [24]. The underuse of mentoring could be due to several factors [17, 25, 26]. A first factor is the lack of teaching time and incentives for teachers [25, 26]. A second factor is the structuring of clinical training in short rotations, which hampers more longitudinal relationships between students and teachers. A third explanation for the underuse of mentoring is a lack of formal mentoring programmes, in combination with a culture in which looking for a mentor is sometimes seen as a sign of weakness, and the inability of students or residents to find mentors by themselves. Under which circumstances is mentoring effective? Successful mentoring relationships are marked by reciprocity, personal connection, mutual respect, shared values and clear expectations. Mentoring is dysfunctional when the mentor takes advantage of the mentee or when the mentor has preconceived ideas about the choices students should make [27]. A mentor should ask questions and help mentees reflect on their choices, instead of advising them what to choose and offering solutions to their problems. By questioning and encouraging reflection the mentor enables the mentee to make their own decisions and arrive at their own solutions. For doctors this can be challenging because in patient care they are expected to offer concrete solutions to their patients [28]. In order to make mentoring effective, a mentee should be active and take ownership of the mentoring by informing the mentor about their (learning) needs and expectations [29]. To do this, a safe environment is essential, an atmosphere in which the mentees feel free to discuss in an open manner their development with their mentor [30].

### Engagement

There is another revolution in a neighbouring discipline that may have great relevance to education. In organisational psychology a large field of study has emerged that researches how workers become engaged [31], a field originally stemming from positive psychology [32]. Engagement is a state of highest satisfaction in employees that is characterized by vitality, dedication and absorption [33]. Engagement is, in a way, the opposite to burnout. Based on abundant empirical work, a simple model proposes how work may lead to work engagement, associated with increased production, or to burnout, associated with production loss [34]. The outcome is mediated by a delicate interaction between job demands, job resources and one’s personal resources [35]. Engagement is associated and promoted through autonomy, self-control, social support and coaching, constant change, diversity, teamwork, interesting challenges, etc. It is the opposite from external control, prescribed tasks, boredom, individuality, etc. Work engagement theory resonates strongly with the work around self-determination and intrinsic or extrinsic motivation [36] that we have already mentioned and also has strong repercussions for education [20].

Our education practices are full of external control, boring tasks (listening to teachers for hours), dull and anxious exams, limited (organized) social support, complete summative exam systems dominating all (poor) learning, etc. We should not be surprised that our learners are so externally motivated or are not engaged in their role as a learner. Teacher-centred programmes are associated with a poorer learning climate and more depressive systems and burnout [37]. The same is true for learners in the workplace [38]. Even in very potentially challenging education environments such as workplaces, inactive learners doing very repetitive learning tasks become disengaged. Learners learn best when they actually participate in the work with their own (supervised) responsibility [39], as part of the health care team where they experience a sense of belonging [40]. Our education should be geared to engagement and excellence, not to fulfilling obligations and minimum standards. It should provide learners challenging tasks. For example, when paper patient problems were replaced by real patient encounters in a problem-based learning setting, the motivation of students was tremendously boosted [41]. Our learners should receive more trust, responsibility and autonomy. In sum, how workers become engaged in a work environment is very similar to how learners become engaged in a learning environment.

### Learning in a social context

Most of the above areas of evidence, perhaps with the exception of collaborative learning, consider learning as an individual activity. In recent years theories have emerged that place learning in a more social context, in which learning from the context of meaningful social relationships and practices is considered to be very important [42]. Situated learning is one of the social cultural learning theories and extends the classic notion of apprenticeship as learning by observation and imitation through to active participation learning in a community of professionals [43]. Social learning theories have inspired quite some medical education researchers to study learning in the workplace and arrive at models of learning [13, 39, 40].

The clinical phase of medical training programmes typically follows the classic apprenticeship-learning model. Students rotate through a set of disciplinary rotations. Creating many transitions in workplace rotations seems to be harmful to learning [21]. The number of disciplines and the time of duration of the attachments is often historically determined and also often a result of fierce debates on the status of individual disciplines. Social learning theories have inspired newer approaches to clinical rotations. Creating learning experiences in which the learner carries clinical responsibilities under appropriate supervision and where the learner is part of the health care team leads to different structuring of these rotations. One example is the recent promotion of longitudinally integrated clerkships where a learner is immersed in a clinical environment for a longer period of time [44–46]. There is more continuity and responsibility in these clerkships than in the shorter traditional versions of them. Research seems to indicate improved learning in academic results, enhanced patient-centredness, greater exposure to normal conditions, and more meaningful relationships with patients and academic mentors [47]. Another example of where sociocultural learning theory is influential is the recent attention to work-based assessment [48]. The intent is to provide more meaningful feedback to the learners from the professionals involved in the workplace [49], to drive self-directed learning through the use of reflection and dialogues with respected supervisors in enduring relationships to guide learning [13]. One may note the clear links to the notions expressed earlier in relation to engagement and mentoring, all pointing to similar directions of development of more optimized learning.

## From theory to practice: do more with less

The above six areas of evidence naturally provide a rather haphazard overview of evidence in education. Even attempts to be comprehensive [50] are not really comprehensive and become quickly outdated. This shows how research in education is evolving. More important than the representativeness of the evidence is the gap between the evidence and educational practice. If we would only take these six more or less arbitrary areas of evidence seriously, we could radically change existing practices! Obviously we are much in favour that education programmes should allow learners to meaningfully work with their knowledge, using challenging tasks, with learners working in cooperation, getting meaningful feedback on their complex tasks. In doing so we should guide and support them in entrusted relationships, and we should give them autonomy, team membership in the academic or clinical community, and we should promote excellence, not minimalism.

We can easily guess the thoughts of the reader. These are expensive suggestions! What is the cost of all this? Who can afford that in times of economic hardship? Our argument would be that we spend our resources incorrectly. In most of our curricula we spend most time and effort actually on information delivery. In the most conventional programmes—probably still the vast majority of programmes in the world—20 to 30 h is easily spent on information transfer or delivery. As is clear from the above, good learning is considerably more than information delivery. Modern technology may provide the answer on the expenditure question. With the above evidence on learning we should stop spending substantial parts of our resources on delivery. We would propose to capture all information delivery on the computer and distribute that through the web. Initiatives such as Modern Online Open Courses (MOOCs) and online academies with online content are conquering the world [51]. Some even fear the destruction of academia [52]. To some extent it is a shame that a professor in for example anatomy in Australia covers the same information as the anatomy professor in Canada. Why don’t we capture the information and construct attractive video material as an addendum or replacement to written materials? Granted, this will take resources. But all the technology is there and is becoming increasingly cheaper and accessible. To save resources we could make this a collective effort. It seems a waste of resources to develop these on our own independently of each other.

Once the information delivery is available, perhaps even publically, we can put all our efforts into supporting the learning process. The quality of programmes will not depend on how to give the best information from the best teachers, but on the way we as teachers are able to scaffold the learning process that maximally facilitates that learning. Then we have time to discuss content in small-scale situations and in close interaction with our learners. We will be able to give appropriate feedback on learner performance, and we will have time to guide and mentor them throughout a programme. Learners will become engaged. Fun will be an essential element for both learners and teachers. So is excellence.

## Discussion

The idea of distributing course content and focussing on student-centred learning is not new. Prober and Heath have called the approach ‘lecture halls without theatres’ [53]. A more common term is ‘flipped classroom’ [54] and we note reports and first attempts on implementing these ideas in health education programmes [55]. These are great initiatives. We need to focus on education strategies that support the learning process of the learner. Strategies that are based on research information and theories that explain why things work in education under which circumstances.

In doing so we should forget about comparing curriculum X versus Y to see which is superior. Instead we should acquaint ourselves with evidence and theory from the educational sciences, perhaps even participate and contribute to the scholarly work. Then we should engage ourselves in creatively designing educational strategies that make optimized translations from theory to education practice. We should exchange our best practices and learn from each other. By doing further research we may actually also advance theory development, which again may shape future practices.

Essential in this approach to education is the research-informed [56] creative design of instructional strategies and the way we implement them. Implementation often determines the ultimate quality of curricula and is often neglected. [57] We have seen implementations of problem-based learning curricula that did not work properly, simply because the implementation failed [58]. Buy-in from faculty about change is key to any success. Research in the educational sciences tells us that teachers’ perceptions on learning and their belief system on learning, partly being unconscious, determines the way they act in education practice [59]. This stems from a wider theory in psychology in planned behaviour [60], often used for changing health-related behaviours [61]. Changing behaviours is a challenging task in any professional field. In the research on Continuous Professional Development, where attempts are being made to convince doctors to use recent evidence, the most common approach of having experts explain what to do is least effective [62]. A multimodal approach closely aligned to the needs of the doctor seems to work best [63]. We would therefore argue that the learning evidence on student learners equally applies to our teachers. Simply by telling them to change will be the best recipe for disaster. We will need to involve them, allow them to experience working in different ways, having a change leader or manager who is entrusted, who coaches, who helps. By doing this well, teachers will also become engaged and this will fuel the change process. We need the same creativity to translate the theory of change [57] to designs of faculty development and change management strategies, while taking the evidence on learning into account.

We hope to have sketched a perspective on the use of evidence in education. We should spend less time and resources on information delivery, but rather on educational strategies that better support the process of learning. We should be cautious doing rather blind comparisons of full curricula. We should seek or contribute to education evidence that explains why educational strategies work and under which conditions. We should use that information to design concrete educational strategies. We have a choice to simply continue based on tradition and intuition, or we could use the evidence to shape our future educational practices. Alternatively, if we do not use evidence in education, than the evidence is no more than fine art in the attic.

## Essentials

- Educational practice and educational research are misaligned.
- Too much effort is given to instruction which is not effective.
- Information delivery to learners should be web-distributed.
- Research evidence provides inspiration for more effective approaches supporting learning.
- Research evidence should be directed on explaining why an education strategy works.
